# Blood pressure control, hypertension phenotypes, and albuminuria: outcomes of the comprehensive Basel Postpartum Hypertension Registry

**DOI:** 10.1038/s41440-025-02191-2

**Published:** 2025-04-25

**Authors:** Leana N. Hotz, Thilo Burkard, Alessandro Rana, Celine A. Wenker, Subeedhja Jalanthiran, Leana Piattini, Noémie Strobel, Viviane Vorster, Zoë G. Menzinger, Sophia Eichler, Christina Schumacher, Michael Mayr, Michael Dickenmann, Irene Hoesli, Olav Lapaire, Beatrice Mosimann, Annina S. Vischer, Thenral Socrates

**Affiliations:** 1 Medical Outpatient Department and Hypertension Centre, ESH Hypertension Centre of Excellence, University Hospital Basel, Basel, Switzerland; 2 Department of Cardiology, University Hospital Basel, Basel, Switzerland; 3 Department of Nephrology, University Hospital Basel, Basel, Switzerland; 4 Department of Obstetrics and Gynecology, University Hospital Basel, Basel, Switzerland

**Keywords:** Cardiovascular risk, Hypertensive disorders in pregnancy, Postpartum hypertension, Blood pressure phenotypes

## Abstract

Postpartum hypertension (PPHT) affects 20% of pregnancies and is strongly correlated to cardiovascular and kidney disease. Most outcome data stems from preeclampsia (PE) neglecting other hypertensive disorders of pregnancy (HDP). This analysis aimed to investigate blood pressure (BP) control, BP phenotypes, therapeutic intensity scores (TIS), and albuminuria across the spectrum of PPHT in the short-medium term.This analysis prospectively followed 370 cases of PPHT. Automated office BP measurements (AOBPM), 24-hour ambulatory BP measurements (24ABPM), TIS and Kidney Disease Improving Global Outcomes (KDIGO) > A2 levels of albumin to creatinine ratio (ACR) were measured at 3 (V3) and 12 (V12) months postpartum. Outcomes were percentage of participants with non-hypertensive AOBPM and awake 24ABPM, whitecoat, and masked hypertension, and an A2 ACR at V3 and V12. The Basel-PPHT cohort consisted of 11.9% (*n* = 44) chronic hypertension, 31.9% (*n* = 118) gestational hypertension, 55.4% (*n* = 205) PE, eclampsia or HELLP, and 18.4% (*n* = 68) de novo PPHT. Antihypertensive medication was prescribed at baseline, V3 and V12 in 85.4% (*n* = 316), 19.2% (*n* = 46), and 20% (*n* = 21). At V12, 9.3% (*n* = 5) with PE, eclampsia, and HELLP vs 31.4% (*n* = 16) of the remaining cohort required antihypertensive medication, *p* = 0.005. Non-hypertensive BP without medication was seen at V3 and V12 in 47.9% (*n* = 103) and 62.4% (*n* = 63), respectively. Albuminuria at baseline, V3 and V12 was 84.9% (*n* = 124), 29.9% (*n* = 63), and 16.9% (*n* = 14) respectively. The Basel-PPHT registry identified undertreatment and persistent albuminuria, despite structured management. Importantly, those without preeclampsia also required stricter controls. Therefore, rigorous follow-ups are crucial for enhancing cardiovascular and renal outcomes in this population.

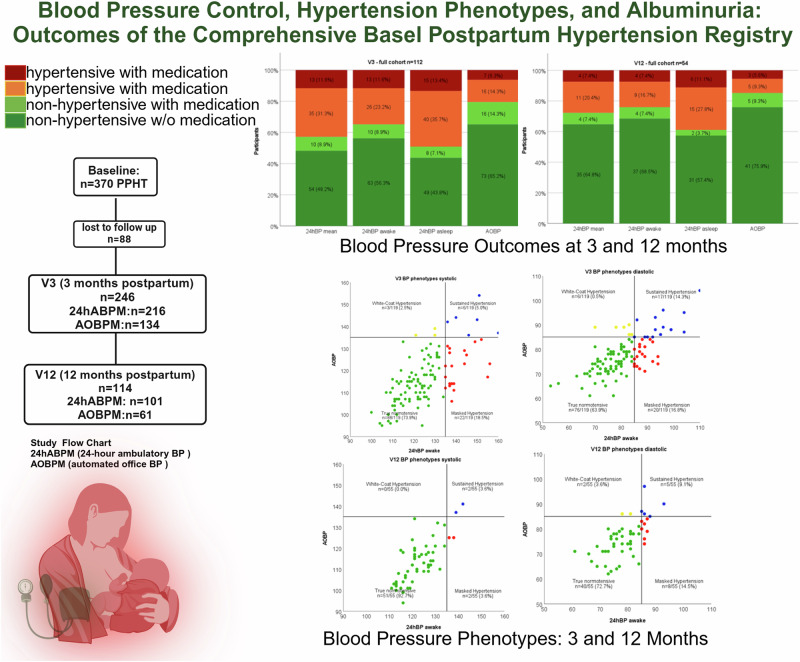

## Introduction

Postpartum hypertension (PPHT), defined as elevated blood pressure after delivery, affects ~20% of pregnancies and its incidence is projected to rise due to advancing maternal age and increasing obesity rates [1–3]. PPHT is a major risk factor for rehospitalization and maternal mortality, necessitating accurate diagnosis prior to discharge [4–7].

PPHT can manifest de novo, with a prevalence of 1 in 10, or be caused by various etiologies including chronic hypertension (CH) or hypertensive disorders of pregnancy (HDP) including gestational hypertension (GH), preeclampsia (PE), eclampsia and HELLP (Hemolysis, Elevated Liver Enzymes, Low Platelets) [8, 9]. While it is understood that blood pressure normalization patterns differ between PE (typically slower normalization due to endothelial damage) and GH (generally rapid normalization), individuals with PPHT—even those with early blood pressure normalization—exhibit a higher risk of developing essential hypertension earlier in life compared to the general population [10, 11]. Although albuminuria typically normalizes within 2 years postpartum in individuals with PE, its trajectory in a heterogeneous PPHT population remains unclear. Existing data on long-term cardiovascular and renal risks associated with HDP are primarily derived from studies focusing on PE, leaving the long-term outcomes of individuals with other forms of PPHT, particularly de novo PPHT, largely unknown [11–15].

This complex and undocumented phase in the medical history of the mother creates a knowledge gap which extends to the immediate postpartum period, where concrete management guidelines and screening protocols for long-term cardiovascular risk factors are lacking [16, 17]. While 24 hour ambulatory blood pressure measurement (24hABPM) is the gold standard for the diagnosis of hypertension, this is rarely implemented in this patient population and specific blood pressure patterns such as nocturnal hypertension, white-coat hypertension (WCH) or masked hypertension (MH) may be missed [18].

This study presents findings from the Basel PPHT Registry, a prospective cohort study designed to provide structured follow-up for individuals with all-cause PPHT. We examined 24-hour ambulatory blood pressure monitoring (24hABPM), automated office blood pressure measurements (AOBPM), Therapeutic Intensity Scores (TIS), and albumin/creatinine ratios as a surrogate marker for target kidney damage at 3 and 12 months. By employing these diagnostic tools within a structured registry, we aim to identify risk factors for persistent hypertension and characterize distinct hypertension phenotypes in individuals with PPHT.

## Methods

### Ethics

The study protocol complies with the Declaration of Helsinki, was approved by the local ethics committee, Ethikkommission Nordwest- und Zentralschweiz (Ethics Commission Northwest and Central Switzerland), (EKNZ 2020-00736), and registered (NCT04690660). Informed consent was obtained from all participants.

### Study procedures, design, definitions

The Basel Postpartum Hypertension Registry (Basel-PPHT) is a single center prospective observational registry based at the University Hospital Basel, Switzerland, as described previously [19]. In brief, the Hypertension Centre of the Medical Outpatient Department and the Department of Obstetrics and Gynecology at the University Hospital Basel are conducting this study in collaboration. After delivery individuals were screened and recruited by the treating physicians and the study team. Treatment and follow-up were based on the local standards of the Hypertension Centre of the Medical Outpatient Department and according to the treating physician.

Eligible for enrollment into the registry were all individuals with preexisting hypertension, HDP and de novo PPHT (defined as blood pressure measurements of systolic ≥140 and/or diastolic ≥ 90 mmHg or the indication of antihypertensive therapy up to 6 weeks after delivery) or individuals on antihypertensive medication after delivery with an age ≥ 18 years [20]. HDP included GH, PE, eclampsia and HELLP, and final diagnoses were made by the treating obstetrician. For the diagnosis of PPHT a minimum of two blood pressure measurements of >139 mmHg systolic and/or >89 mmHg were required, measured during routine vital signs monitoring on nursing rounds.

The study’s exclusion criteria included: younger than 18 years of age, lack of consent to participate in the study, or lack of general understanding regarding the study and consent.

Following inclusion, data were gathered from a structured interview with a combination of self-reported medical history and the hospital’s electronic documentation system and entered into an online database.

Key visits of the study were baseline, defined as inclusion into the study after obtaining informed consent, landmark visit 3 (V3, ~3 months after delivery) and landmark visit 12 (V12, ~12 months after delivery). Annual landmark visits were planned till 5 years after delivery but were not part of this interim analysis. When clinically indicated additional visits were scheduled between landmark visits at the discretion of the treating physician.

**Fig. 1:**
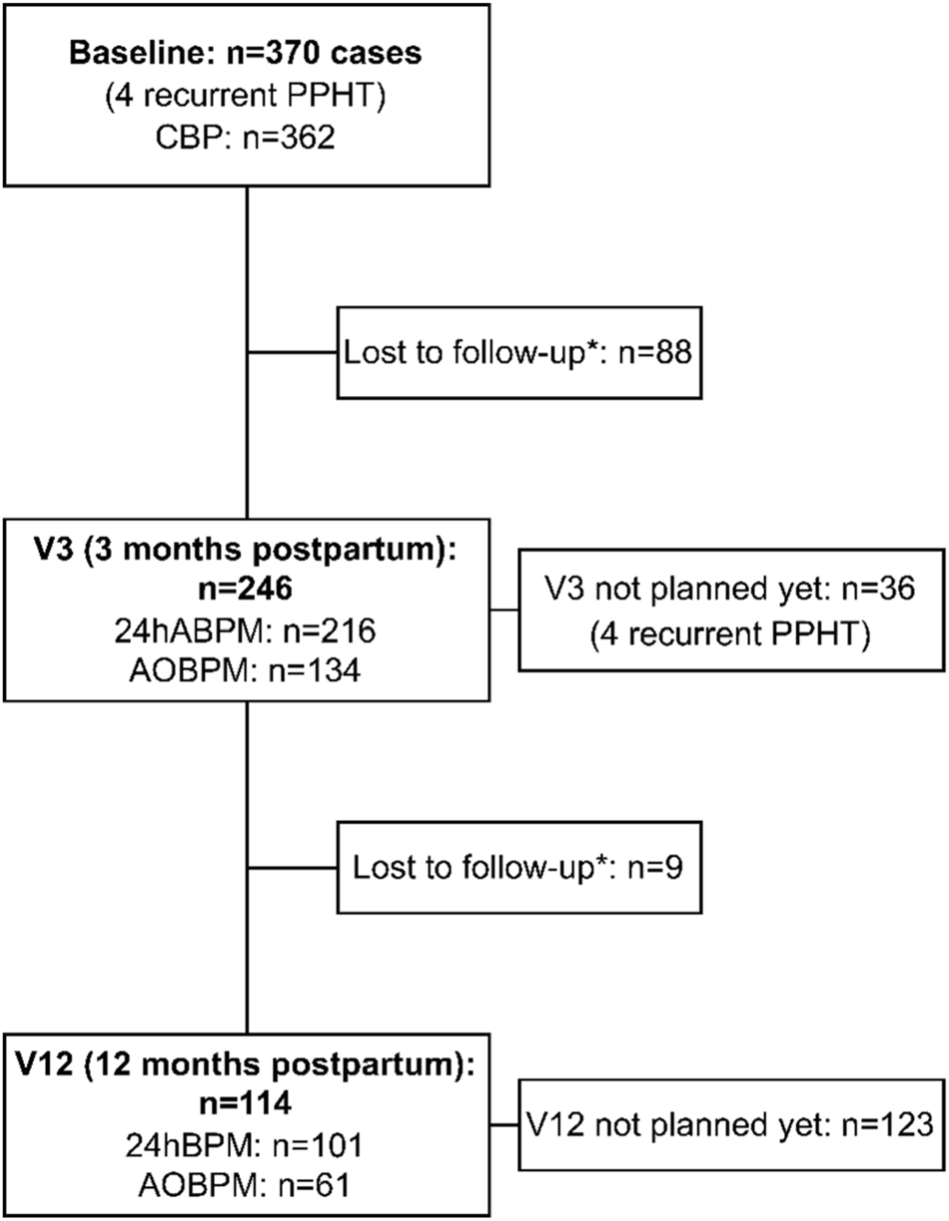
Flow Chart of all Participants included in the Basel-PPHT Registry. CBP clinic blood pressure, 24hABPM 24-hour ambulatory blood pressure measurement, AOBPM automated office blood pressure measurement. *lost to follow up through to canceled appointment, non-appearance, or further care provided by the family doctor

Enrollment started in June 2020. This interim analysis analyzed data from baseline to clinical visits between 04.06.2020 and 31.10.2023, which included a total of 366 participants and 370 pregnancies (Fig. 1).

#### Blood pressure measurement procedures

##### In-hospital clinic blood pressure and after discharge

In-hospital clinic blood pressure (CBP) was monitored during routine clinic visits from the nursing staff at the Obstetrics Departement. Automatic unattended measurements were taken with a Welch Allyn blood pressure device. These measurements were automatically sent to the patient’s electronic chart and examined by the treating physician. CBP was defined as the mean of the first three measurements.

After discharge patients were treated as clinically indicated, either with scheduled outpatient visits, an asynchronous telemonitoring system or a programmed spreadsheet. An unattended AOBPM was taken during all outpatient visits that took place at the Hypertension Clinic of the Medical Outpatient Department and at the landmark visits a 24hABPM was performed.

##### 24-hour Ambulatory Blood Pressure Measurement (24hABPM)

Routine 24hABPM was scheduled at V3 and V12. Three various clinically validated devices were implemented for 24ABPM including: Mobil-O-Graph, Mobil-O-Graph PWA or Spacelabs 90217 A [21–23]. The devices measured the BP every 20 min from 8:00 to 22:00 and every 30 min during the night. Individual participant logs were used to define awake and asleep times. Corresponding analysis software (Spacelabs Healthcare Inc, USA and Mobil-O-Graph, IEM GmBH, Aachen, Germany) analyzed the values after the measurement and calculated the mean systolic and diastolic 24-h, awake and asleep BP values. As recommended in the European Society of Hypertension (ESH) 2023 and European Society of Cardiology (ESC) 2024 guidelines, hypertensive mean 24hABPM was defined as mean systolic/diastolic ≥130/ ≥ 80 mmHg and awake and asleep were defined as ≥135/ ≥ 85 mmHg and ≥120/ ≥ 70 mmHg, respectively [24–26].

##### Unattended automated office blood pressure measurement

To measure the AOBPM patients were instructed to sit upright in a chair with uncrossed legs and both feet on the ground alone in a quiet room. The correct cuff was chosen based on the circumference of the arm and was positioned at the level of the heart. After performing a test measurement, to check if the device was working properly, trained nursing staff initiated the measurement sequence and left the room. Devices were programmed to automatically take three measurements after 5, 7, and 9 min. A clinically validated Welch Allyn Connex® Spot Monitor was used, which applies the SureBP® measurement technique by Welch Allyn [27]. The mean of these three measurements was calculated automatically by the device. Non-hypertensive AOBPM was defined as systolic/diastolic BP < 135/ < 85 mmHg [28].

##### Blood pressure phenotypes

White-coat hypertension (WCH) refers to the discrepancy between elevated office blood pressure and lower home or ambulatory BP, primarily reflecting a response to anxiety during office visits. WCH occurs in ~30% of pregnant individuals and is more prevalent than in the general population. Despite a better maternal and neonatal prognosis compared to CH, the risk of PE and preterm birth is significantly higher in white-coat hypertension than in normotension [29]. Masked hypertension (MH) is characterized by normal office BP with elevated out-of-office BP measurements in untreated patients. Here we used awake 24 hABPM to determine if a white-coat effect or MH was present.

##### Therapeutic intensity score

Therapeutic intensity score was calculated to determine the intensity of antihypertensive drug treatment. It is defined as the sum of all antihypertensive drug doses taken by the patient divided by the maximum dose of each antihypertensive drug, e.g., a half dose of drug 1 and a full dose of drug 2 results in a TIS of 1.5. The maximum dose of each antihypertensive drug is listed in the Therapeutic Intensity Score Table S1 in the supplement.

##### Routine blood and urine sampling

Predefined laboratory values of blood and urine assessments at the visits were recorded out of the electronic health records, when ordered by the treating physician.

Kidney Disease Improving Global Outcomes (KDIGO) ≥ A2 levels of albumin to creatinine ratio (ACR) were used to define relevant albuminuria [30].

### Statistical methods

The distribution of continuous variables was determined using skewness, kurtosis and visual inspection of the histograms. Continuous data were reported as mean ± standard deviation (SD), median interquartile range (IQR) and compared using the unpaired *t*-test and Mann-Whitney-*U*-Test, as appropriate Categorical variables are described as counts (percent) and compared using chi-square test or fisher’s exact tests, as appropriate. The entire cohort was analyzed as described above.

Prespecified groups for the analysis were (1) individuals with diagnosis of PE, eclampsia and HELLP and (2) individuals with PPHT without PE, eclampsia or HELLP. For example, a participant with GH starting at week 27 and PE at week 34 would be assigned to group 1.

Statistical analysis was performed using SPSS software, with a *p* value of <0.05 set to indicate statistical significance.

## Results

### Baseline characteristics

A total of 366 participants and 370 pregnancies were enrolled in the registry from 06/2020-10/2023. The mean age was 34 (±5.4) years. Mean weight after delivery was 83.2 (±19.8) kg and before pregnancy 71.3 (±18.0) kg. Caucasian ethnicity was reported in 83%, (*n* = 307) of the cohort. The most common comorbidity was a history of hypertension 11.6%, (*n* = 43), followed by diabetes mellitus 10.5%, (*n* = 39) and thyroid disease 8.9%, (*n* = 33). The mean systolic blood pressure at baseline was 136.2 (±13.5) mmHg and diastolic 85 (±8.4) mmHg. Antihypertensive treatment was prescribed in 85.4% with median TIS of 0.5 (0.3–1.0). The median TIS was significantly higher in individuals with PE 0.8 (0.5–1.0) compared to individuals without PE 0.5 (0.3–0.8), *p* < 0.001. The most prescribed drug was enalapril 73.7%, *n* = 233 followed by metoprolol 63.3%, *n* = 200. In terms of PPHT aetiology 11.9%, (*n* = 44) had CH, 31.9%, (*n* = 118) GH 55.4%, (*n* = 205) PE, eclampsia or HELLP and de novo PPHT was seen in 18.4%, (*n* = 68). The mean number of days between birth and baseline visit was 7.8 (±36.3) days. The baseline characteristics from the full cohort and the prespecified groups are shown in Table 1.

**Table 1:** Baseline Characteristics

Characteristics *n* (%)	Full Cohort	PreeclampsiaEclampsia, HELLP *n* = 205 (55.4)	HDP, CH and de novo PPHT *n* = 165(44.6)	*p* value
Age (years) Mean (SD) Median (IQR)	*n* = 369 34.0 (±5.4) 34 (30–37)	*n* = 204 33.8 (±5.5) 34 (30–37)	*n* = 165 34.1 (±5.3) 34 (30.5–38)	0.511
Height (cm), Mean (SD) Median (IQR)	*n* = 368 164.9 (±5.9) 165 (160–169)	*n* = 204 164.4 (±5.5) 164(160–168)	*n* = 164 165.5 (±6.3) 165 (160–170)	0.17
Weight before Pregnancy (in kg), Mean (SD) Median (IQR)	*n* = 357 71.3 (±18.0) 67 (59–80)	*n* = 194 68.5 (±14.8) 65.5 (59–75)	*n* = 163 74.5 (±20.8) 68 (58–87)	0.032
Weight at Baseline (kg), Mean (SD) Median (IQR)	*n* = 347 83.2 (±19.8) 79 (70–92)	*n* = 196 80.1 (±17.4) 78 (69–88)	*n* = 151 87.3 (±22.1) 82 (70–102)	0.006
BMI Preconception (kg/m2), Mean (SD) Median (IQR)	*n* = 358 26.1 (±6.3) 24.4(21.5–29.1)	*n* = 194 25.3 (±5.2) 24.1(21.8–8.0)	*n* = 164 27.1 (±7.4) 24.9 (21.4–31.8)	0.0142
Baseline CBP systolic, Mean (SD) Median (IQR)	*n* = 362 136.2 (±13.5) 136 (126–146)	*n* = 202 136.6 (±13.0) 136 (126–147)	*n* = 160 135.8 (±14.1) 135.5(127–145)	0.611
Baseline CBP Diastolic, Mean (SD) Median (IQR)	*n* = 362 85.0 (±8.4) 85 (79–90)	*n* = 202 85.2 (±8.4) 85 (79–91)	*n* = 160 84.8 (±8.1) 85 (79–89)	0.506
Antihypertensive Medication at Baseline *n* (%)^a^	316/370 (85.4)	175/205 (85.4)	141/165 (85.5)	1.0
- Labetalol	89/370 (28.2)	49/205 (28.0)	40/165 (28.4)	1.0
- Metoprolol	200/370 (63.3)	123/205 (70.3)	77/165 (54.6)	0.012
- Nifedipine	65/370 (20.6)	36/205 (20.6)	29/165 (20.6)	1.0
- Enalapril	233/370 (73.7)	133/205 (76.0)	100/165 (70.9)	0.449
- other medication	2/370 (0.6)	1/205 (0.6)	1/165 (0.7)	1.0
- Lisinopril	2/370 (0.6)	1/205 (0.6)	1/165 (0.7)	1.0
- Methyldopa	3/370 (1.0)	1/205 (0.6)	2/165 (1.4)	0.588
Baseline TIS Mean (SD) Median (IQR)	*n* = 315 0.7 (±0.5) 0.5 (0.3–1.0)	*n* = 174 0.8 (±0.5) 0.8 (0.5–1.0)	*n* = 141 0.5 (±0.4) 0.5 (0.3–0.8)	<0.001
Ethnicity *n* (%)				
Caucasian	307/369 (83.2)	168/204 (82.4)	139/165 (84.2)	0.676
Asian	17/369 (4.6)	11/204 (5.4)	6/165 (3.6)	0.465
Black or African Origin	24/369 (6.5)	11/204 (5.4)	13/165 (7.9)	0.398
Middle Eastern	11/369 (3.0)	7/204 (3.4)	4/165 (2.4)	0.761
More than one Ethnicity	3/369 (0.8)	3/204 (1.5)	0/165 (0.0)	0.256
Unknown	8/369 (1.9)	4/204 (2.0)	3/165 (1.8)	1.000
Comorbidities, *n* (%)^a^				
- None	236/370 (63.8)	142/205 (69.3)	94/165 (57.0)	0.017
- Cardiovascular Disease	8/370 (2.2)	2/205 (1.0)	6/165 (3.6)	0.146
- Renal Disease	4/370 (1.1)	1/205 (0.5)	3/165 (1.8)	0.328
- Thyroid Disease	33/370 (8.9)	20/205 (9.8)	13/165 (7.9)	0.585
- Other	32/370 (8.6)	15/205 (7.3)	17/165 (10.3)	0.354
Cardiovascular Risk Factors, *n* (%)				
- Lack of Exercise	63/370 (17.0)	31/205 (15.1)	32/165 (19.4)	0.330
- Preconception obesity (BMI > 30 kg/m2)	79/370 (21.4)	38/205 (18.5)	41/165 (24.8)	0.161
- Arterial Hypertension	43/370 (11.6)	9/205 (4.4)	34/165 (20.6)	<0.001
- Dyslipidemia	6/370 (1.6)	4/205 (2.0)	2/165 (1.2)	0.696
- Family History of Cardiovascular Disease	190/370 (51.4)	102/205 (49.8)	88/165 (53.3)	0.531
- Diabetes	39/370 (10.5)	21/205 (10.2)	18/165 (10.9)	0.866
- History of Smoking	138/359 (38.4)	68/196 (34.7)	70/163 (42.9)	0.127
- None	76/370 (20.5)	46/205 (22.4)	30/165 (18.2)	0.365

Data presented as mean(±SD), median (IQR), *n* (%)

Mean(±SD) compared using the unpaired *t*-test

Median interquartile range (IQR) Mann-Whitney-*U*-Test

*p* values are shown next to means or medians based on the appropriate distribution

Categorical variables are described as counts (percent) and compared using chi-square test or fisher’s exact tests, as appropriate

Self-reported by the patients or taken from electronic medical records

^a^More than one choice possible

### Cardiovascular risk factors

The most common cardiovascular risk factor was a positive family history of cardiovascular disease, reported in 51.4%, (*n* = 190) of the cohort. 38.4%, (*n* = 138) had a history of smoking, of which 6.4%, (*n* = 23) were active smokers. Preconception obesity was present in 21.4%(*n* = 79) of the cohort (Table 1). Additional cardiovascular risk factors are shown in Supplementary Table S2. A family history of PE was reported in 8.1%(*n* = 29), and pregnancy induced hypertension in 5.6%(*n* = 20) as seen in Table S2 (Supplementary Information).

### Delivery characteristics

The most common mode of delivery was cesarean section with 68.6%, *n* = 254 followed by vaginal birth with 23%, *n* = 85 and vacuum delivery with 8.1%, *n* = 30. Mean weeks of gestation at delivery was 36.7 (±3.5) weeks, with a significant lower number in the PE group (*p* < 0.001). The mean weight of the newborn was 2814 (±863.3) grams. The weight was significant lower in the PE group as well (*p* < 0.001). Intrauterine growth restriction was observed in 21%, (*n* = 77) of the individuals, in 28.1%, (*n* = 57) in the group with PE and 12.3%, *n* = 20 in the other group (*p* < 0.001). All delivery characteristics are shown in Table S3 in the Supplementary Information.

### Blood pressure outcomes and control at 3 months

Out of the 246 individuals, 216 had a 24hABPM at V3. Mean 24h systolic and diastolic blood pressure was at V3 121.7 (±11.0) mmHg and 77.3 (±8.6) mmHg respectively. Mean awake systolic and diastolic blood pressure at V3 was 124.6 (±11.6) and 80.5 (±9.0) mmHg respectively. Mean asleep systolic and diastolic blood pressure was 114.5 (±11.2) and 69.7(±8.4) mmHg respectively. All mean BP values are shown in Table S4 in the appendix.

Antihypertensive medication was prescribed in 46 participants with a median TIS of 0.5 (0.3–1.0). TIS in individuals without PE 0.5 (0.3–0.9) was the same than in individuals with PE 0.5 (0.3–1.4), but not statistically significant (*p* = 0.812). All data according to antihypertensive medication and the TIS are shown in (Tables 2 and 3).

**Table 2:** Antihypertensive Medication and Therapeutic Intensity Score (TIS) V3 and V12

	Full Cohort (*n* = 370)	Preeclampsia, eclampsia, HELLP (*n* = 205)	HDP, CH and de novo PPHT excluding PE, eclampsia and HELLP (*n* = 165)	*p* value
V3 *n* (%) Antihypertensive Medication	46/239 (19.2%)	20/131 (15.3)	26/108 (24.1)	0.086
V3 TIS Mean (SD) Median (IQR)	0.7 (±0.6) 0.5 (0.3–1.0)	0.6 (±0.5) 0.5 (0.3–0.9)	0.8 (±0.7) 0.5 (0.3–1.4)	0.256
V12 *n* (%) Antihypertensive Medication	21/105 (20%)	5/54 (9.3%)	16/51 (31.4%)	0.005
V12 TIS Mean (SD) Median (IQR)	0.6 (±0.4) 0.5 (0.3–1.0)	0.6 (±0.4) 0.5 (0.1–1.0)	0.6 (±0.4) 0.5 (0.3–1.0)	0.867

Data presented as mean (±SD), median (IQR); *n* (%)

Mean(±SD) compared using the unpaired *t*-test

Median interquartile range (IQR) Mann-Whitney-*U*-Test

*p* values are shown next to means or medians based on the appropriate distribution

**Table 3:** Antihypertensive medication and therapeutic intensity score (TIS) V3 and V12

	Full Cohort (*n* = 370)	Preeclampsia, eclampsia, HELLP (*n* = 205)	HDP, CH and de novo PPHT excluding PE, eclampsia and HELLP (*n* = 165)	*p* value
V3 *n* (%) Antihypertensive medication	46/239 (19.2%)	20/131 (15.3)	26/108 (24.1)	0.086
V3 TIS mean (SD) median (IQR)	0.7 (±0.6) 0.5 (0.3–1.0)	0.6 (±0.5) 0.5 (0.3–0.9)	0.8 (±0.7) 0.5 (0.3–1.4)	0.812
V12 *n* (%) Antihypertensive medication	21/105 (20%)	5/54 (9.3%)	16/51 (31.4%)	0.005
V12 TIS mean (SD) median (IQR)	0.6 (±0.4) 0.5 (0.3–1.0)	0.6 (±0.4) 0.5 (0.1–1.0)	0.6 (±0.4) 0.5 (0.3–1.0)	0.386

Data presented as mean (±SD), median (IQR); *n* (%)

Mean(±SD) compared using the unpaired *t*-test

Median interquartile range (IQR) Mann-Whitney-*U*-Test

*p* values are shown next to means or medians based on the appropriate distribution

AOBPM were available in 134 individuals with a mean of 116.6 (±11.6) mmHg systolic and 77.4 (±7.6) mmHg diastolic.

**Fig. 2:**
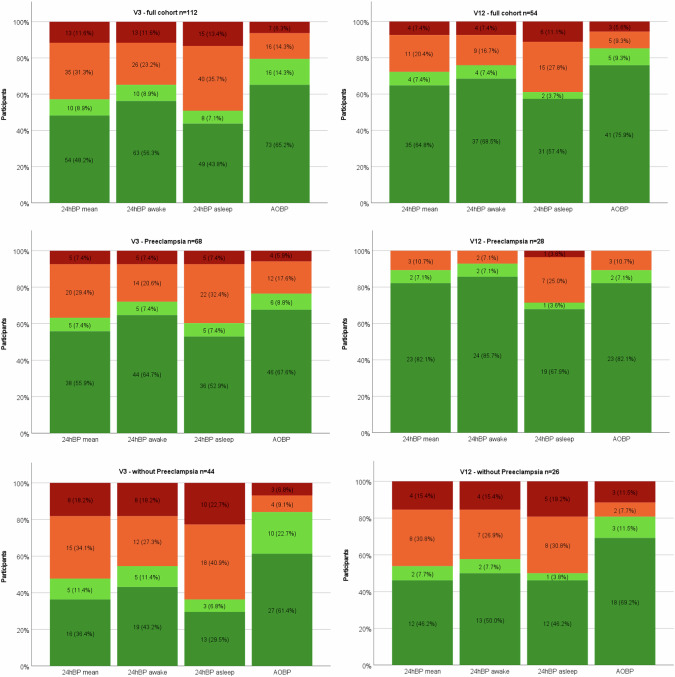
Blood pressure outcomes at 3 and 12 months for the full cohort and the subgroups of patients with and without preeclampsia.  hypertensive with medication  hypertensive without medication  non-hypertensive with medication  non-hypertensive without medication. *Stacked column chart comparing the different blood pressure measurement types (24hABP mean, awake, asleep and AOBPM) at V3 and V12 for the full cohort and the subgroups of patients with and without preeclampsia. Only participants, who had all four different BPM were included in the figure. Columns show the different BP control: hypertensive with medication, hypertensive without medication, non-hypertensive without medication and non-hypertensive with medication*

Out of the 216 individuals with an awake 24hABPM, 55.1% (*n* = 119) were non-hypertensive without antihypertensive treatment, 8.8% (*n* = 19), were non-hypertensive with treatment, 10.6% (*n* = 23), were hypertensive with treatment and 24.5% (*n* = 53), were hypertensive without treatment. Blood pressure control in individuals with all four methods of blood pressure measurement is shown in Fig. 2. Mean BP at V3 and V12, and the number of participants being treated with antihypertensive medication and if blood pressure control was achieved is shown in Table S6 and Table S7, respectively in the Supplementary Material.

### Blood pressure outcomes at 3 months in patients with or without preeclampsia

Out of the 112 patients at V3, who had a 24hABPM and an AOBPM, 68 (60.7%) had PE, eclampsia or HELLP and 44 (39.3%) CH, GH or de novo PPHT without manifest PE. In the PE group, awake 24hABPM was non-hypertensive in 64.7% (*n* = 44), without medication, non-hypertensive with medication in 7.4% (*n* = 5), hypertensive without medication in 20.6% (*n* = 14) and hypertensive with medication in 7.4% (*n* = 5). In the non-PE group only 43.2% (*n* = 19) were non-hypertensive without medication, 11.4% (*n* = 5) non-hypertensive with medication, 27.3% (*n* = 12) were hypertensive without medication and 18.2% (*n* = 8) were hypertensive with medication. All results are shown in Fig. 2.

### Blood pressure outcomes and control at 12 months

At V12, mean systolic and diastolic 24hBP was 119.3 (±8.5) mmHg and 75.5 (±6.9) mmHg, respectively. Awake systolic and diastolic BP was 122.5 (±8.5) and 78.9 (±7.1) mmHg, respectively. Asleep systolic and diastolic BP was 111.7 (±10.8) and 67.4 (±8.3) mmHg, respectively. Mean systolic and diastolic AOBPM was 112.8 (±10.3) mmHg and 75.4 (±7.2) mmHg, respectively. All mean BP values are shown in Table 2 in the Appendix. 21 participants were under antihypertensive treatment with a median TIS of 0.5 (0.3–1.04). The TIS was the same in individuals with and without PE as shown in Table 2. 65.3% (*n* = 66) of the individuals with a 24ABPM were non-hypertensive without antihypertensive treatment, 11.9% (*n* = 12) were non-hypertensive with treatment, 6.9% (*n* = 7) were hypertensive with treatment and 13.9% (*n* = 14) were hypertensive without treatment. The BP control at V12 is shown in Fig. 2 and in Table S7 in the appendix.

### Blood pressure outcomes at 12 months in patients with or without preeclampsia

At V12, 24hABPM was available in 54 patients, 28 (51.9%) having PE, eclampsia, or HELLP and 26 (48.1%) with a CH, GH, or de novo PPHT without manifest PE. Within the awake 24hABP readings in the PE group, 85.7% (*n* = 24) of patients were non-hypertensive without medication, 7.1% (*n* = 2) were non-hypertensive with medication, 7.1% (*n* = 2) were hypertensive without medication and no patients were hypertensive with medication. In those without PE, 50% (*n* = 13) of were non-hypertensive without medication, 7.7% (*n* = 2) were hypertensive with medication, 26.9% (*n* = 7) were hypertensive without medication, and 15.4% (*n* = 4) were hypertensive with medication. These results are presented in Fig. 2.

### Blood pressure phenotypes at 3 and 12 months

At V3 and V12 a 24hABP awake and an AOBPM were available in 119 and 55 individuals respectively. MH was present at V3 and V12 in 20.2% (*n* = 24) and 12.7% (*n* = 7) of individuals, respectively. Regarding the different groups at V3, 12.5% (*n* = 9) individuals with PE had a MH compared to 31.9% (*n* = 15) of individuals without PE. At V12 MH was seen in 3.4% (*n* = 1) and 23.1% (*n* = 6), respectively.

WCH at V3 was seen in 5.9% (*n* = 7) of the full cohort, in 8.3% (*n* = 6) with PE and 2.1% (*n* = 1) without PE. At V12 WCH was present in 3.6% (*n* = 2), all these individuals had PE.

**Fig. 3:**
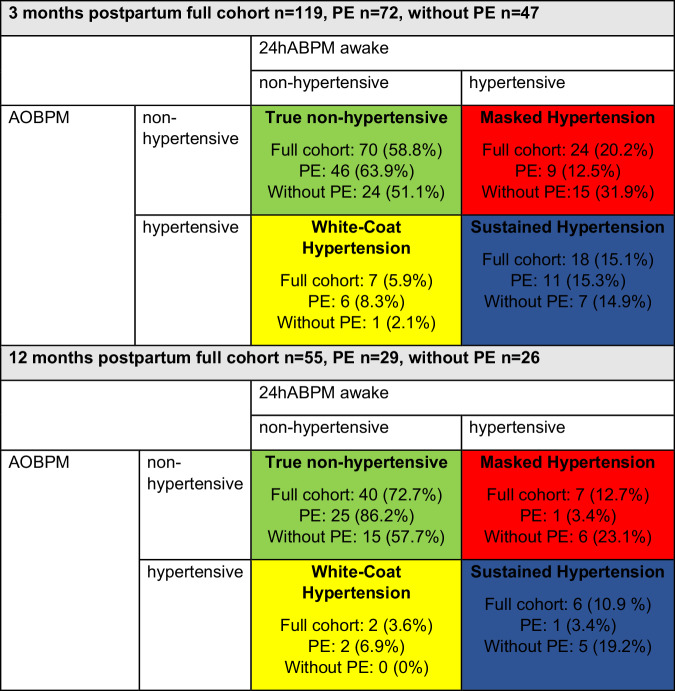
Blood Pressure Phenotypes: True Non-hypertensive, Sustained Hypertension, White-Coat Hypertension, Masked Hypertension. *Comparison of AOBPM and 24ABPM at 3 and 12 months postpartum to identify blood pressure phenotypes (true non-hypertensive, masked hypertension, white-coat hypertension and sustained Hypertension). Data is presented as n (%)*

At V3 and V12 true normotension was seen in 58.8% (*n* = 70) and 72.7% (*n* = 40) respectively. Sustained hypertension, high blood pressure in the 24hABPM awake and the AOBPM, was seen in 15.1% (*n* = 18) and 10.9% (*n* = 6) of individuals, respectively. Different blood pressure phenotypes at V3 and V12 (Fig. 3). In addition, we analyzed the systolic and diastolic blood pressure separately, as shown in Fig. S1 in the Supplementary Information.

### Albuminuria

The prevalence of albuminuria at baseline, V3, and V12 was 84.9% (*n* = 124), 29.9% (*n* = 63) and 16.9% (*n* = 14) respectively. All cause HDP/PPHT baseline mean (±SD) ACR was 89.3(±366) mg/mmol. Quantitatively at V3 and V12 it was 5.8(±17.1) mg/mmol and 2.9(±9.5) mg/mmol respectively. In those with PE ACR at baseline was seen in 92.2% (*n* = 83), at V3 35.6% (*n* = 42) and at V12 19.1% (*n* = 9). Albuminuria in those without PE at baseline was 73.2% (*n* = 41), at V3 22.6% (*n* = 21) and at V12 13.9% (*n* = 5).

The median ACR ratio in the group with PE at V3 was higher with 1.9 (0.9–4.1) mg/mmol compared to 1.6 (0.8–2.8) mg/mmol in the group without PE (*p* = 0.115). At V12 the median ACR of those with PE was 1.4 (0.6-2.1) mg/mmol compared to the other group 1.0 (0.7–1.9) mg/mmol (*p* = 0.825). Median baseline ACR in the PE group was significantly higher at 21 (7.8-65.1) mg/mmol compared to 5.5 (2.7-17.7) mg/mmol (*p* = 0.001).

**Fig. 4:**
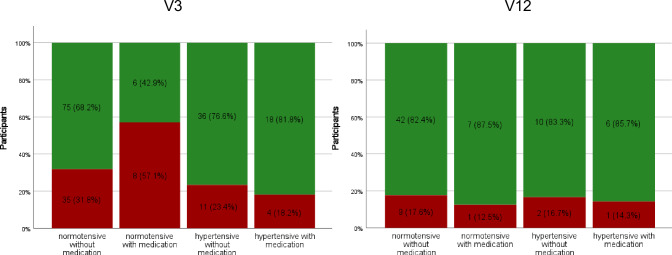
Prevalence of albuminuria at V3 and V12, 3 and 12 months respectively.  ACR < KDIGO A2  ACR ≥ KDIGO A2. Stacked column chart comparing the prevalence of albuminuria in participants with normotensive blood pressure with and without medication and those with hypertensive awake 24ABP blood pressure with and without medication at V3 and V12. Relevant levels of albumin to creatinine ratio (ACR) albuminuria was based on the classification: Kidney Disease Improving Global Outcomes (KDIGO) ≥ A2

The prevalence of albuminuria at V3 in non-hypertensive individuals without medication was 31.8% (*n* = 35), controlled hypertension with medication 57.1% (*n* = 8), hypertensive without medication 23.4% (*n* = 11), and in hypertensive with medication 18.2% (*n* = 4) (Fig. 4 and Table S4).

At V12 albuminuria was seen in 9/51 (17.6%) of non-hypertensive individuals without medication, 1/8 (12.5%) in non-hypertensive individuals with medication, 2/12 (16.7%) in hypertensive individuals without medication and 1/7 (14.3%) in hypertensive individuals with medication (Fig. 4 and Table S4).

## Discussion

Our study provides several observations and insights regarding blood pressure outcomes and patterns of individuals with postpartum hypertension. Notably, patients with PE or related disease vs. individuals without PE, were represented nearly 1:1 in our cohort.

First- In general, normalization of mean 24hABP at 3 and 12 months postpartum was seen in ~50% and 60% of participants, respectively. Blood pressure control was achieved in ¾ of the total cohort, including those on antihypertensive medication. However, a surprisingly a high number of participants were hypertensive and after cessation of medication which was based on clinical indication. Non-hypertensive AOBPM at 3 and 12 months postpartum were seen in 8 and 9 out of 10 participants, respectively. Normalization of awake BP was higher than asleep BP. 24hABPM 3 and 12 months postpartum showed a high prevalence of nocturnal hypertension, which may be explained by disturbed sleep in the setting of caring for a newborn. Therefore, the validity of the asleep 24hABPM needs to be interpreted with caution in terms of clinical decision-making.

Second- Blood pressure was more frequently inadequately controlled in participants without manifest PE, eclampsia or HELLP. Postpartum BP control at 12 months was achieved in 90% of participant with PE and only in 50% of participants without PE. Of the 90% of participants with PE, the vast majority were non-hypertensive without antihypertensive medication.

Third- At 3 and 12 months postpartum 20.2% and 12.7% of the cohort had MH, indicating a high risk of undertreatment of these individuals. White-coat hypertension was present only in ~5% at 3 and 12 months postpartum, probably due to the use of AOBPM, which reduces the WCH effect compared to non-standardized measurements [31]. Individuals without PE had a higher prevalence of MH (23.1% vs 3.4% at V12) and a lower risk for WCH (0% vs 6.9% at V12) compared to individuals with PE. To investigate the blood pressure phenotypes, we considered only the awake 24hBP, as the 24hBP asleep may overestimate hypertension as described above.

Fourth- At 1-year postpartum, albuminuria normalized in most participants, but an elevated albumin/creatinine ratio persisted in ~1 out of 6 participants. At 3 months almost 1 in 3 participants had persistent albuminuria. Albuminuria after 12 months was present in 19.1% of participants with PE and 13.9% in participants without PE. Although the number was higher in individuals with PE, this was not statistically significant. Remarkably, 12 months postpartum, the prevalence of albuminuria was almost the same regardless of BP control or treatment.

From this data we can conclude that, reaching blood pressure control during the 1st year after delivery in individuals with PPHT remains a challenging task. A relevant percentage of participants were not adequately controlled after seemingly indicated cessation of medication, which may indicate that antihypertensive treatment was prematurely down titrated. As Lee et al. demonstrated follow up after withdrawal of antihypertensive medication for up to 6 months is important in young patients due to rebound hypertension [32].

Individuals with GH, CH and new onset of PPHT without PE were at a higher risk for persistent hypertension. This is important in terms of physician and patient awareness regarding postnatal management especially because generally only PE patients are considered high risk for rehospitalization due to uncontrolled blood pressure and future cardiovascular disease. The TIS at baseline was significantly higher in the PE group 0.8 and 0.5 (<0.001) respectively. In individuals with PE the TIS shows a downward trend between Baseline, V3 and V12. In individuals without PE the TIS remains unchanged up to V12, which reflects the diagnosis of CH.

The significant prevalence of MH underscores the need for 24-hour ambulatory blood pressure monitoring in the postpartum period. Future guidelines should specify the optimal timing for this assessment, with our data suggesting that three months postpartum is an appropriate time to adjust medication.

Two previous studies examining blood pressure phenotypes in individuals with PE reported MH rates of 11.6% and white-coat hypertension rates of 17.9% at 6–12 weeks postpartum, decreasing to 17.5% and 9.5%, respectively, 1 year after delivery [33, 34]. These findings indicate a substantial risk of both under- and overtreatment in individuals with PE. Our results reveal an even higher risk of MH in individuals without PE, suggesting that 24hABPM should be implemented for both groups. White-coat hypertension occurred in less than 5% of our cohort at 12 months, lower than reported in other national registries, likely due to the use of unattended office blood pressure monitoring, which is known to reduce white-coat hypertension [35]. These findings could be explained due to the implementation of unattended office BPM instead of normal office BPM. This method of measuring BP is known to reduce WCH as described above.

A relevant number of patients in our cohort had albuminuria. This again reiterates that tighter BP control and management in the first postpartum year is essential. In our cohort, even participants with adequately controlled blood pressure exhibited evidence of kidney damage. Berks et al. have shown a higher resolution of proteinuria 3 months postpartum with only 14% of individuals still having proteinuria and after 2 years only 2%. However, a different cut-off was used with 0.3 g/d and KDIGO categories was not investigated [12]. Spaan et al. has shown that the resolution of albuminuria can take up to 2 years after delivery [36]. Therefore, longer follow-up of these patients and further studies are required to identify risk factors for persistent albuminuria.

Our findings are consistent with other studies in the field of HDP, however most of the cohorts studied only included individuals with PE. For example, Berks et al. showed that 39% of individuals with PE are still hypertensive at 3 months and 18% at 2 years postpartum [12]. Girsberger et al. published data on preeclamptic individuals from the University hospital of Basel in 2018, demonstrating a 20% incidence of persistent hypertension and proteinuria 6 months after delivery [37]. This group did not perform 24hABPM, but their findings are similar to our AOBPM in individuals with PE.

Evidence is insufficient on pregnancy outcomes in individuals with CH, GH and de novo PPHT without manifest PE are lacking. Interestingly, de Novo PPHT, is being recognized as more frequent than previously determined, with new data showing a 10% incidence in normotensive pregnancies [8]. Individuals with de novo PPHT are typically not considered at increased risk of developing CH, as their elevated blood pressure postpartum is often attributed to factors such as pain, volume overload, stress and medication [9]. However, our study shows a significantly high number of individuals without PE with hypertensive blood pressure 12 months postpartum. Therefore, regardless of etiology, individuals with PPHT are at a high risk for undertreatment probably due to underestimating their risk and therapeutic inertia.

### Strength and limitations

To our knowledge this is first study to enroll and analyze all causes of PPHT including CH, HDP, and de novo PPHT. This fills a gap in knowledge due to the fact that most outcome studies focus mainly on PE. In addition, we implemented a 24hABPM, the gold standard for the diagnosis of arterial hypertension and were able to identify different blood pressure phenotypes.

Most importantly this cohort of women with PPHT were treated using an interdisciplinary approach with transition of care after discharge to a European Hypertension Excellence Centre with internal medicine specialist, cardiologists, nephrologists, and obstetricians working closely together. In centers with complex multimorbid patients, secondary causes of hypertension, certain hypertensive phenotypes, and adverse pregnancy outcomes; specialized follow-up is merited. Increasing data indicates that in these situations, postpartum management from a multidisciplinary team or physicians with training in obstetric medicine, improves long term maternal outcomes [38, 39].

A limitation of our study was that the discharge diagnosis was made by the treating obstetrician, sometimes without critical information such as proteinuria or postpartum development of PE. In terms of ACR, we did not have data regarding how many participants had a pathological albumin/creatinine ratio before pregnancy, which would have affected the interpretation of albuminuria. Additionally, there was often an overlap in diagnosis. During the SARS-CoV-2 pandemic inpatient visits were not always possible, and many visits were made by telephone, which explains the lower number of AOBPM compared to 24hBPM.

## Supplementary information

Table S1

Table S2

Table S3

Table S4

Table S6

Table S7

Figure S1
