# Supplementary material for: Blood pressure control, hypertension phenotypes, and albuminuria: outcomes of the comprehensive Basel Postpartum Hypertension Registry
Source: Hypertens Res. 2025 Apr 25;48(7):2095–107. doi: 10.1038/s41440-025-02191-2 (PMC12229887; doi:10.1038/s41440-025-02191-2)
Supplement: Supplementary file 1 — Table S1 [file 41440_2025_2191_MOESM1_ESM.docx]

**Table S1. Therapeutic Intensity Score**

**Maximum doses of Antihypertensive Medication.**

| Antihypertensive Medication | Maximum Dose in mg |
| --- | --- |
| Labetalol hydrochloride (Trandate) | 2400 ^22^ |
| Atenolol | 100 ^23,24,25^ |
| Metoprolol succinate (Belok zoc ret.) | 200 ^25^ |
| Nifedipine (Adalat CR ret./ Nifedipin Mepha ret.) | 90 ^26^ |
| Amlodipine | 10 ^24,25,27^ |
| Enalapril maleate (Reniten mite) | 40 ^24,25,28^ |
| Perindopril arginine (Coversum) | 10 ^24,29,30,31^ |
| Methyldopa (Aldomet) | 3000 ^32^ |
| Lisinopril (Zestril) | 40 ^24,25,33^ |
