# Supplementary material for: Blood pressure control, hypertension phenotypes, and albuminuria: outcomes of the comprehensive Basel Postpartum Hypertension Registry
Source: Hypertens Res. 2025 Apr 25;48(7):2095–107. doi: 10.1038/s41440-025-02191-2 (PMC12229887; doi:10.1038/s41440-025-02191-2)
Supplement: Supplementary file 2 — Table S2 [file 41440_2025_2191_MOESM2_ESM.docx]

**Table S2: Additional Baseline Characteristics***

|  | **Full cohort**  **(n=370)** | **Preeclampsia, eclampsia, HELLP**  **(n=205)** | **HDP, CH and de novo PPHT Excluding PE, Eclampsia and HELLP**  **(n=165)** | **p-value** |
| --- | --- | --- | --- | --- |
| **Current Smoking Status, n (%)** |  |  |  |  |
| - Active | 23/359 (6.4) | 11/196 (5.6) | 12/163 (7.4) | 0.523 |
| - Never | 221/359 (61.6) | 128/196 (65.3) | 93/163 (57.1) | 0.127 |
| - Quit | 115/359 (31.1) | 57/196 (29.1) | 58/163 (35.6) | 0.212 |
| **Medical History of Previous Pregnancies,**  **n (%) *** |  |  |  |  |
| - Uncomplicated | 66/370 (17.8) | 36/205 (17.6) | 30/165 (18.2) | 0.892 |
| - Gestational Hypertension | 16/370 (4.3) | 5/205 (2.4) | 11/165 (6.7) | 0.069 |
| - Preeclampsia | 31/370 (8.4) | 16/205 (7.8) | 15/165 (9.1) | 0.708 |
| - Postpartum Hypertension | 15/370 (4.1) | 2/205 (1.0) | 13/165 (7.9) | <0.001 |
| **Family History Related to Hypertensive Disorders in Pregnancy (First Degree), n (%)*** |  |  |  |  |
| - Family History of Pregnancy Induced Hypertension | 20/358 (5.6) | 10/195 (5.1) | 10/163 (6.1) | 0.818 |
| - Family History of Preeclampsia | 29/358 (8.1) | 20/195 (10.3) | 9/163 (5.5) | 0.121 |
| - Family History of Postpartum Hypertension | 10/358 (2.8) | 4/195 (2.1) | 6/163 (3.7) | 0.522 |
| - Unknown | 130/358 (36.3) | 75/195 (38.5) | 55/163 (33.7) | 0.379 |
| - None | 169/358 (47.2) | 86/195 (44.1) | 83/163 (50.9) | 0.204 |

data presented n (%). data was self-reported or was taken from electronic medical records* more than one choice possible
