# Supplementary material for: Blood pressure control, hypertension phenotypes, and albuminuria: outcomes of the comprehensive Basel Postpartum Hypertension Registry
Source: Hypertens Res. 2025 Apr 25;48(7):2095–107. doi: 10.1038/s41440-025-02191-2 (PMC12229887; doi:10.1038/s41440-025-02191-2)
Supplement: Supplementary file 3 — Table S3 [file 41440_2025_2191_MOESM3_ESM.docx]

**Table S3: Type of Delivery and Newborn Characteristics**

|  | **Full cohort**  **(n=370)** | **Preeclampsia, eclampsia, HELLP**  **(n=205)** | **HDP, CH and de novo PPHT excluding PE, eclampsia and HELLP**  **(n=165)** | **p-value** |
| --- | --- | --- | --- | --- |
| **C-Section, n (%)** | 254/370 (68.6) | 149/205 (72.7) | 105/165 (63.6) | 0.071 |
| **Vaginal, n (%)** | 85/370 (23.0) | 43/205 (21.0) | 42/165 (25.5) | 0.322 |
| **Vacuum, n (%)** | 30/370 (8.1) | 13/205 (6.3) | 17/165 (10.3) | 0.183 |
| **Forceps, n (%)** | 1/370 (0.3) | 0/205 (0.0) | 1/165 (0.6) | 0.446 |
| **Gestational Age at Birth**  **Mean (SD)**  **Median (IQR)** | n=369  36.7 (±3.5)  37 (35-39) | n=205  35.7 (±3.8)  36 (34-38) | n=164  38.0 (±2.4)  38 (37-40) | <0.001 |
| **Intrauterine growth restriction n (%)** | 77/366 (21.0) | 57/203 (28.1) | 20/163 (12.3) | <0.001 |
| **Newborn Birth Weight (g)**  **Mean (SD)**  **Median (IQR)** | n=339  2814 (±863.3)  2990 (2280-3425) | n=185  2526 (±911.8)  2590 (1950-3215) | n=154  3160 (±652.7)  3265 (2774-3590) | <0.001 |

data is presented as mean (+/- SD), median (IQR); n (%), weight in grams

mean(±SD) compared using the unpaired t-test

median interquartile range (IQR) Mann-Whitney-U-Test

p values are shown next to means or medians based on the appropriate distribution
