# Supplementary material for: Blood pressure control, hypertension phenotypes, and albuminuria: outcomes of the comprehensive Basel Postpartum Hypertension Registry
Source: Hypertens Res. 2025 Apr 25;48(7):2095–107. doi: 10.1038/s41440-025-02191-2 (PMC12229887; doi:10.1038/s41440-025-02191-2)
Supplement: Supplementary file 4 — Table S4 [file 41440_2025_2191_MOESM4_ESM.docx]

**Table S4: Prevalence and extent of albuminuria at Baseline, V3 and V12**

|  | **Full cohort** | **Preeclampsia, eclampsia, HELLP** | **HDP, CH and de novo PPHT excluding PE, eclampsia and HELLP** | **p-value** |
| --- | --- | --- | --- | --- |
| Baseline n (%)  albuminuria  ACR mg/mmol  median (IQR) | 124/146 (84.9)  12.7 (4.5-45.3) | 83/90 (92.2)  21.0 (7.8-65.1) | 41/56 (73.2)  5.5 (2.7-17.7) | <0.001 |
| V3 n (%)  albuminuria  ACR mg/mmol  median (IQR) | 63/211 (29.9)  1.6 (0.8-3.8) | 42/118 (35.6)  1.9 (0.9-4.1) | 21/93 (22.6)  1.6 (0.8-2.8) | 0.863  0.115 |
| V12 n (%)  albuminuria  ACR mg/mmol  median (IQR) | 14/83 (16.9)  1.2 (0.6-2) | 9/47 (19.1)  1.4 (0.6-2.1) | 5/36 (13.9)  1.0 (0.7-1.9) | 0.825 |

Data presented as median (IQR), n (%)

albumin to creatinine ratio (ACR)
