# Supplementary material for: Blood pressure control, hypertension phenotypes, and albuminuria: outcomes of the comprehensive Basel Postpartum Hypertension Registry
Source: Hypertens Res. 2025 Apr 25;48(7):2095–107. doi: 10.1038/s41440-025-02191-2 (PMC12229887; doi:10.1038/s41440-025-02191-2)
Supplement: Supplementary file 5 — Table S6 [file 41440_2025_2191_MOESM5_ESM.docx]

**Table S6. Blood Pressure Outcomes at V3**

|  | Systolic mmHg | Diastolic mmHg |
| --- | --- | --- |
| n=215  24hBP Mean (SD) | 121.7 (±11.0) | 77.3 (±8.6) |
| n=216  24hBP Awake Mean (SD) | 124.6 (±11.6) | 80.5 (±9.0) |
| n=206  24hBP Asleep Mean (SD) | 114.5 (±11.2) | 69.7 (±8.4) |
| n=134  AOBPM Mean (SD) | 116.6 (±11.6) | 77.4 (±7.6) |
|  |  |  |
| **24 h Mean <130/80**  **(n=215*)** | **Non-Hypertensive**  **n=121/215 (56.3%)*** | **Hypertensive**  **n=94/215 (43.7%)*** |
| **Medication n=42** | 17 (7.9%) | 25 (11.6%) |
| **No Medication n=171** | 103 (47.9%) | 68 (31.6%) |
| **24 h Awake < 135/85 (n=216*)** | **Non-Hypertensive**  **n=139/216 (64.4%)*** | **Hypertensive**  **n=77/216 (35.6%)*** |
| **Medication n=42** | 19 (8.8%) | 23 (10.6%) |
| **No Medication n=172** | 119 (55.1%) | 53 (24.5%) |
| **24 h Asleep < 120/70 (n=206*)** | **Non-Hypertensive**  **n=102/206 (49.5%)*** | **Hypertensive**  **n=104/206 (50.5%)*** |
| **Medication n=40** | 14 (6.7%) | 26 (12.6%) |
| **No Medication n=164** | 87 (42.2%) | 77 (37.4%) |
| **AOBPM <135/85**  **(n=134*)** | **Non-Hypertensive**  **n=105/134 (78.4%)** | **Hypertensive**  **n=29/134 (21.6%)*** |
| **Medication n=29** | 19 (14.2%) | 10 (7.5%) |
| **No Medication n=104** | 86 (64.2%) | 18 (13.4%) |

data is presented as mean (+/- SD), median (IQR); n (%).

* data missing regarding antihypertensive medication in 2 patients
