# Supplementary material for: Blood pressure control, hypertension phenotypes, and albuminuria: outcomes of the comprehensive Basel Postpartum Hypertension Registry
Source: Hypertens Res. 2025 Apr 25;48(7):2095–107. doi: 10.1038/s41440-025-02191-2 (PMC12229887; doi:10.1038/s41440-025-02191-2)
Supplement: Supplementary file 6 — Table S7 [file 41440_2025_2191_MOESM6_ESM.docx]

**Table S7. Blood Pressure Outcomes at V12**

|  | Systolic mmHg | Diastolic mmHg |
| --- | --- | --- |
| n=101  24hBP Mean (SD) | 119.3 (±8.5) | 75.5 (±6.9) |
| n=101  24hBP Awake Mean (SD) | 122.5 (±8.5) | 78.9 (±7.1) |
| n=101  24h Asleep Mean (SD) | 111.7 (±10.8) | 67.4 (±8.3) |
| n=61  AOBP Mean (SD) | 112.8 (±10.3) | 75.4 (±7.2) |
|  |  |  |
| **24 h Mean <130/80**  **(n=101*)** | **Non-Hypertensive**  **n=74/101 (73.3%)** | **Hypertensive**  **n=27/101 (26.7)*** |
| **Medication n=19** | 11 (10.9%) | 8 (7.9%) |
| **No Medication n=80** | 63 (62.4%) | 17 (16.8%) |
| **24 h Awake < 135/85**  **(n=101*)** | **Non-Hypertensive**  **n=78/101 (77.2%)** | **Hypertensive**  **n=23/101 (22.8%)*** |
| **Medication n=19** | 12 (11.9%) | 7 (6.9%) |
| **No Medication n=80** | 66 (65.3%) | 14 (13.9%) |
| **24 h asleep < 120/70**  **(n=100*)** | **Non-Hypertensive**  **n=65/100 (65.0%)** | **Hypertensive**  **n=35/100 (35.0%)*** |
| **Medication n=19** | 7 (7.0%) | 12 (12.0%) |
| **No Medication n=79** | 58 (58%) | 21 (21.0%) |
| **AOBP <135/85**  **(n=61)** | **Non-Hypertensive**  **n=53/61 (86.9%)** | **Hypertensive**  **n=8/61 (13.1%)** |
| **Medication n=10** | 7 (11.5%) | 3 (4.9%) |
| **No Medication n=51** | 46 (75.4%) | 5 (8.2%) |

data presented as mean (±SD), median (IQR); n (%),

* data missing in 2 patients
