# Supplementary material for: Blood pressure control, hypertension phenotypes, and albuminuria: outcomes of the comprehensive Basel Postpartum Hypertension Registry
Source: Hypertens Res. 2025 Apr 25;48(7):2095–107. doi: 10.1038/s41440-025-02191-2 (PMC12229887; doi:10.1038/s41440-025-02191-2)
Supplement: Supplementary file 7 — Figure S1 [file 41440_2025_2191_MOESM7_ESM.pdf]

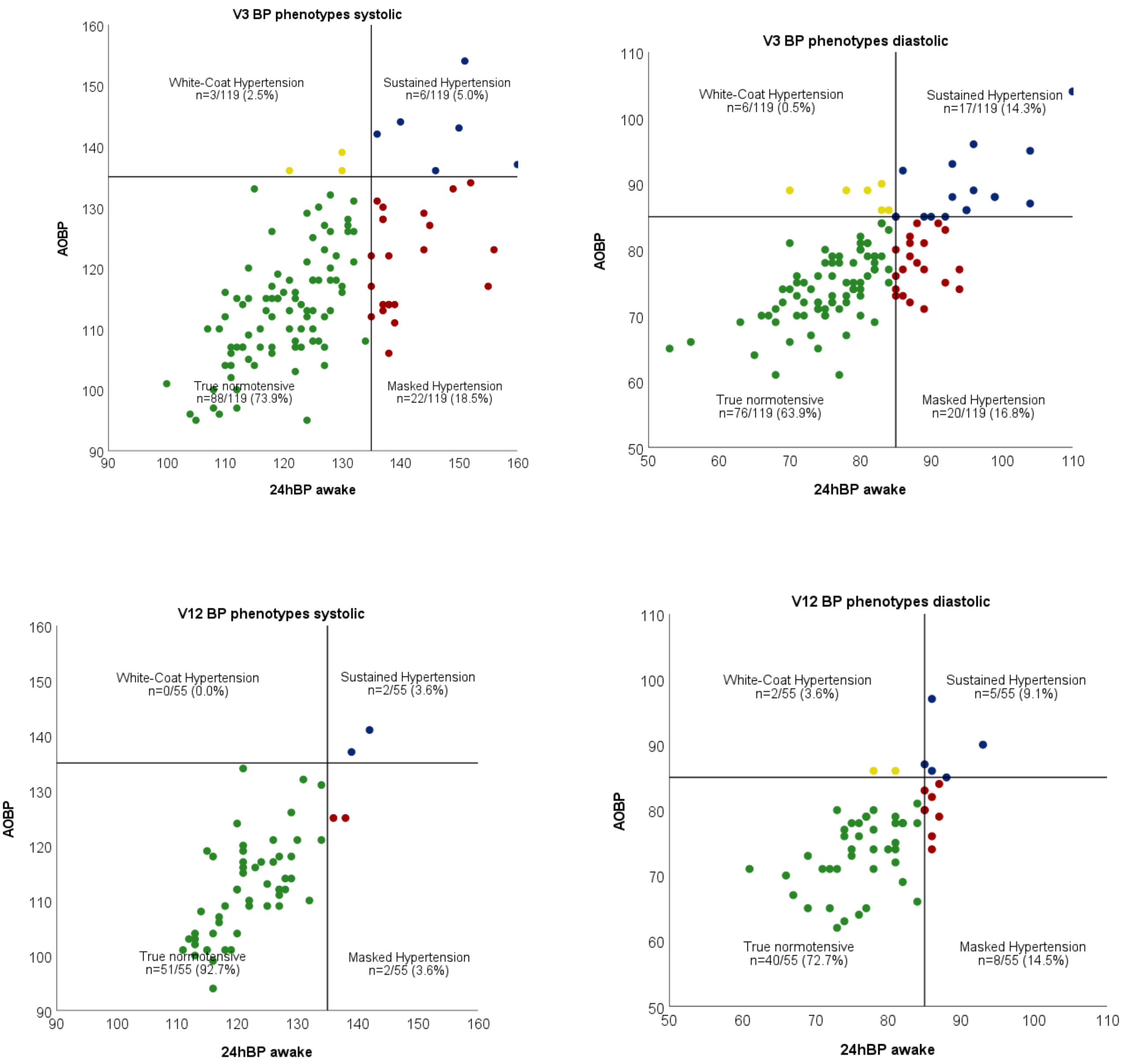

Figure S1: Blood Pressure Phenotypes: divided into Systolic and Diastolic

Comparison between AOBPM and 24hBP awake in a scatter plot. Systolic and diastolic BP presented separately. Non-hypertensive AOBPM and 24hBP was defined as BP < 135/<85 mmHg. True non-hypertensive in green, sustained hypertension in blue, white coat hypertension in yellow and masked hypertension is seen in red.
